# Evaluation of rational use of veterinary drugs especially antimicrobials and anthelmintics in Bishoftu, Central Ethiopia

**DOI:** 10.1186/s13104-015-1466-4

**Published:** 2015-09-28

**Authors:** Takele Beyene, Dagnachew Endalamaw, Yonas Tolossa, Ashenafi Feyisa

**Affiliations:** Department of Biomedical Sciences, College of Veterinary Medicine and Agriculture, Addis Ababa University, P.O.Box 34, Bishoftu, Ethiopia; Department of Veterinary Teaching Hospital, College of Veterinary Medicine and Agriculture, Addis Ababa University, P.O.Box 34, Bishoftu, Ethiopia

**Keywords:** Evaluation, Veterinary drugs, Rational use, Prescribing practices, Ethiopia

## Abstract

**Background:**

Rational use of drugs in veterinary medicine has numerous benefits, such as increasing efficacy, decreasing the potential adverse effects, reducing risk of drug residue and combating development of microorganism’s drug resistance.

**Methods:**

A retrospective study with the aim of evaluating the current rational use of veterinary drugs was conducted at college of veterinary medicine and agriculture veterinary teaching hospital and Ada district veterinary clinic, central Ethiopia. One thousand eight hundred and nineteen animal patients’ encounters were randomly selected for the study from prescription papers and prescription registration books retrospectively.

**Results:**

The average number of drugs prescribed per encounter was 1.23 with maximum of five. The percentage of encounters in which antimicrobials and anthelmintics was prescribed were 54.4 % (1216/2235) and 38.9 % (869/2235), respectively. The percentages of drugs prescribed by generic name and from essential veterinary drug list were 90.1 % (2014/2235) and 99.7 % (2229/2235), respectively. The most commonly prescribed antimicrobials and anthelmintics were oxytetracycline 1016 (45.5 %), penicillin and streptomycin combination 168 (7.5 %), sulfa drugs 23 (1.0 %), and albendazole 732 (32.8 %) and ivermectin 137 (6.1 %). Among the 1819 animal-patient encounters, only 57 % (n = 1037) of the prescriptions were written adequately, 43 % (n = 782) incorrectly prescribed and 1179 cases of the adequately specified prescription were tentatively diagnosed. For 656 (53.9 %) and 233 (26.8 %) inadequately specified cases antimicrobials and anthelmintics were prescribed, respectively. Antibiotics were prescribed irrationally for cases which were tentatively diagnosed as parasitic 21.6 % (n = 262) and viral to prevent secondary bacterial complications 6.0 % (n = 73). Among all patients that were admitted to veterinary clinics, 96.6 % (1757) were treated empirically without getting correct laboratory-supported diagnosis. Chi Square test for trend analysis showed a statistically significant association between irrational drug usage and year (p = 0.000).

**Conclusions:**

The findings had shown problems in generic prescribing, incorrect diagnosis, and non-availability of standard veterinary treatment guideline and drug formulary in the study area. Therefore, veterinary drugs, specially, antimicrobial agents should be judiciously used; and a wide scale study to safeguard the public from drug residual effects and antimicrobial resistance development is recommended.

## Background

Rational use of drugs is based on the use of right drug, right dosage and right cost which is well reflected in the world health organization (WHO) definition: “Rational use of drugs requires that patients receive medications appropriate to their clinical needs, in doses that meet their own individual requirements for an adequate period of time, at the lowest cost to them and their community” [1].

Now, in the clinical practice of human and veterinary medicine throughout the world large amount of antibiotics are used. Equally, many scientists intensively work on discovery and synthesis of new drugs with broader antimicrobial spectrum, stronger action and more satisfactory safety profile. Most failures during antimicrobial therapy may occur when the pathogenic microorganism is unknown and combination of two or more drugs administered empirically. To avoid these mistakes, clinically confirmed, effective antimicrobial combinations should be used [2]. Globally, more than half of all medicines are prescribed, dispensed or sold improperly, and 50 % of human patients fail to take them correctly. This is more wasteful, expensive and dangerous, both to the health of the individual patient and to the population as a whole that magnifies the problem of misuse of antimicrobial agents [3].

Irrational use of drugs in veterinary medicine as well as the need for control of their use becomes even bigger problem when used on food producing animals. In this case, there is the possibility that minimal quantities of drugs and their metabolites (residues) which remain in edible tissues or in animal products (meat, milk, eggs, honey) induce certain harmful effects in humans as potential consumers of such food [4]. When drugs are used to improve the productivity of food animals that are intended for human consumption, then there is possibility for producing adverse effects on humans. To prevent this risk, it is necessary to use drugs rationally, i.e., to use them only when they are really indicated, in the right way, at the right time, in the right dose and respecting withdrawal period. Also, it is necessary to regularly control sensitivity to antimicrobial agents and regulate residue of antimicrobial agents commonly used in veterinary practice [2, 5].

Over use of antimicrobials [6] and anthelmintics [7] in veterinary practice, for both food producing and companion animals, favours the development of both intrinsic or acquired antimicrobial and anthelmintic resistance. Acquired resistance develops due to widespread and irrational use of drugs while intrinsic resistance is a result of inherent structural or functional characteristics, which allows tolerance of a particular drug or antimicrobial class. Antimicrobial/anthelmintic drug resistance is a growing problem; and indeed developing new drugs may not be the solution for this problem. Some of the common causes that contribute to the development of antimicrobial resistance are unnecessary use of antimicrobial drugs, inappropriate dose, inadequate duration of therapy, use of irrational antimicrobial fixed dose drug combinations [8].

In humans, assessments of drug use patterns with the WHO drug use indicators are becoming increasingly necessary to promote rational drug use. These indicators are now widely accepted as a global standard for problem identification and have been used in developing countries [9, 10]. In Ethiopia, a survey conducted on human subjects at hospitals located in different regions of the country revealed the presence of irrational drug use [11–13]. However, in veterinary practice, there is no report on rational use of veterinary drugs in central Ethiopia in particular and in the country in general. Hence, the present study was designed to evaluate the rational use of veterinary drugs and to compare magnitudes of different drugs commonly used for the treatment of animal diseases in the college of veterinary medicine and agriculture veterinary teaching hospital (CVMA-VTH) and Ada district veterinary clinic, central Ethiopia.

## Methods

### Study area and period

The study was conducted from November 2013 to June 2014 in CVMA-VTH and at Ada district veterinary clinic, Bishoftu, central Ethiopia, which is located at 45 km South East of Addis Ababa. Bishoftu is situated at 9°N latitude and 4°E longitude and an altitude of 1850 meter above sea level in the central high lands of Ethiopia. Farmers near to Bishoftu town practice a mixed crop-livestock farming system [14].

### Study design

A retrospective and cross-sectional survey was designed to assess rational drug use and to compare commonly used drugs for treatment of animal diseases at CVMA-VTH and Ada district veterinary clinic. The samples were selected using a systematic random sampling method, and the sampling unit was animal patient encounters at CVMA-VTH and Ada district veterinary clinic for the treatment of acute and/or chronic illness.

### Study population

The study was conducted between November 2013 to June 2014 on food animals/patients (cattle, sheep, goats and chicken of all ages and sex groups) that were admitted to CVMA-VTH and Ada district open-air veterinary clinics and treated with drugs. All other non-food animals (e.g., pets and equines) and animal patients that were admitted to veterinary clinics but did not receive any medicines were excluded from the study.

### Data collection

Data was collected on prescribing indicators retrospectively by using both patient cards and prescription papers in CVMA-VTH and case registration books in Ada district veterinary clinic. The specific data necessary to measure the prescribing indicators was recorded for each animal patient encounter and entered into an ordinary prescribing indicator form. For this particular study, 1819 prescriptions that contain the animal’s characteristics (age, sex, breed, body condition, clinical signs and symptoms observed), disease diagnosis (name, empiric or physical clinical examination and confirmatory laboratory tests used), prescribed drugs (type, naming [generic or brand], number of drugs prescribed, route of administration, duration of treatment, availability in the national veterinary drug list), prescriber’s signature, level of education and years of experiences were collected retrospectively from more than 20,000 prescriptions written for the last 5 years (from January 01, 2009 to December 31, 2013). The availability of both veterinary treatment guidelines and national veterinary drug list (EVDL) in the clinic was also observed. Accordingly, evaluation of rational use of veterinary drugs was made based on generic prescription, and antimicrobials and anthelmintics prescribed for tentatively diagnosed clinical cases.

### Data analysis

All data in the ordinary prescribing indicator recording form were entered into Microsoft Excel spread sheet (version 2010) and imported and analysed using SPSS (Version 20). Means, median (range) and frequencies (percentage) were used to describe patients’ characteristics. The Chi Square trend test was used to check whether there was a linear trend in rational drug use across the years and the year 2009 was used as the reference period. All statistical tests were two sided and P values ≤0.05 was considered significant.

#### Prescribing indicators

There was no available guideline for prescribing indicators used in veterinary medicine. As a result, the WHO prescribing indicators were used in this study [15]. The indicators were pretested and slightly modified to match with clinical practice in veterinary medicine so that they could be used easily to provide accurate data. The final versions of the pretested indicators are:

1. The average number of drugs prescribed per encounter was calculated by dividing the total number of different drug products prescribed with the number of encounters surveyed to measure the degree of poly pharmacy. Fixed combinations of drugs prescribed for one health problem were counted as one;
2. Percentage of drugs prescribed by generic name was calculated by dividing the number of drugs prescribed by generic name with total number of drugs prescribed, multiplied by 100 to measure the tendency of prescribing by generic name;
3. Percentage of encounters in which antimicrobials, anthelmintics, and other drugs prescribed was calculated by dividing the number of patient encounters in which drug was prescribed with the total number of encounters surveyed, multiplied by 100 to measure the overall use of commonly overused (irrationally prescribed) and costly forms of drug therapy;
4. Percentage of drugs prescribed from national veterinary drug list of Ethiopia (EVDL) was calculated by dividing number of products prescribed which are in veterinary drug list with the total number of drugs prescribed, multiplied by 100 to measure the degree to which the practices conform to a national drug policy as indicated in the EVDL of Ethiopia [16];
5. Chi square was used to measure the linear trend in rational and irrational drug use across the years. Rational use of veterinary drugs means sick animals receive medications appropriate to their clinical needs, in doses that meet their own individual requirements, for an adequate period, and at the lowest cost [1, 17] whereas irrational use of drug means misuse of drugs by the patient (i.e., patients receive medications inappropriate to their clinical needs, under or over dosing that meet their own individual requirements, and for inadequate period) [1]. Accordingly, evaluation was made for rational use of veterinary drugs based on generic prescription, and appropriate prescription of antimicrobials and anthelmintics prescribed for tentatively diagnosed sick animals appropriate to their clinical needs.

### Ethical considerations

The study was granted an exemption from requiring ethical approval from the College of Veterinary Medicine and Agriculture Institutional Research and Review Board Committee. The researchers got permission for access to data from the College of Veterinary Medicine and Agriculture and the Ada District Agricultural Office. Confidentiality of the patients’ data and prescribers was maintained by using unique code.

## Results

A total of 1819 patient cards/prescription papers and casebook were assessed from both CVMA-VTH (912) and Ada district veterinary clinic (907). The retrospective study showed that 2235 drug products were prescribed, and the average number of drugs per prescription was 1.23 with a maximum of five drugs. The total number of drugs prescribed by generic name was 2014 (90.1 %). Almost all drugs were prescribed (n = 2229, 99.7 %) according to the EVDL.

The rational drug use evaluation has shown that antimicrobials, anthelmintics, antimicrobial with anthelmintic combinations, antimicrobial with other drugs combinations, and anthelmintic with other drugs combinations were prescribed (Table 1). Out of the total 2235 drugs prescribed, 1216 (54.4 %) antimicrobials, 869 (38.9 %) anthelmintics, and 150 (6.7 %) other drugs (acaricides, vitamins, gastrointestinal stimulants and anti-inflammatory drugs) were prescribed. The most commonly prescribed antimicrobials and anthelmintics were oxytetracycline 1016 (45.5 %), penicillin–streptomycin fixed combination 168 (7.5 %), sulfa drugs (sulfadimidine and sulphametoxazole-trimethoprim fixed combination) 23 (1.0 %), and albendazole 732 (32.8 %) and ivermectin 137 (6.1 %) (Table 2).

**Table 1:** Prescribing indicators at CVMA-VTH and Ada district veterinary clinic from 2009 to 2013

Prescribing indicator	Total	Average/percent
Number of drugs per encounter	2235	1.23
Encounters with antimicrobials	1216	66.8 %
Encounters with anthelmintics	869	47.8 %
Encounters with antimicrobials-anthelmintics combination	333	18.3 %
Encounters with others^a^	150	8.2 %
Encounters with antimicrobials-others combination^a^	75	4.1 %
Encounters with anthelmintic-others combination^a^	53	2.9 %
Encounters with antimicrobials-anthelmintic-others combination^a^	22	1.2 %
Drug prescription by generic name	2014	90.1 %
Drug prescription from EVDL	2229	99.7 %

*EVDL* national veterinary drug list of Ethiopia

^a^Others (acaricides, vitamins, gastrointestinal stimulants and anti-inflammatory drugs)

**Table 2:** Drugs commonly prescribed in CVMA-VTH and Ada district clinic from 2009 to 2013

Drug preparations	Frequency	Percentage (%)
Antimicrobials		
Oxytetracycline	1016	45.5
Penstrep	168	7.5
Sulfa drugs^a^	23	1.0
Penicillin + Cloxacilin	5	0.2
Chloramphenicol	4	0.2
Subtotal	1216	54.4
Anthelmintics		
Albendazole	732	32.8
Ivermectin	137	6.1
Subtotal	869	38.9
Others		
Acaricides^b^	68	3.0
Multivitamin	56	2.5
Gastrointestinal drugs^c^	22	1.0
Dexamethasone	4	0.2
Subtotal	150	6.7
Total	2235	100

^a^Sulfadimidine, Trimethoprim-sulphametoxazole combination

^b^Diazinon and/or malathion

^c^Indigestion powders, magnesium sulphate and mineral oils

Among the total 1819 patient encounters, only 57 % (n = 1037) of the prescription were specified adequately while 43 % (n = 782) were not. From the adequately specified prescription, a total of 1179 diseases were tentatively diagnosed and 335 (27.5 %) of antimicrobials were prescribed irrationally (empiric treatment without confirming the presence of microbial agents) to treat these diseases, where 21.5 % (n = 262) and 6.0 % (n = 73) of them were used for parasitic cases and to prevent secondary bacterial complications in case of viral cases, respectively. For the total 782 (43 %) cases which were not specified adequately, 656 (53.9 %) of antimicrobials and 233 (26.8 %) of anthelmintics were prescribed (Table 3).

**Table 3:** Common animal diseases/conditions diagnosed based on clinical signs and symptoms in CVMA-VTH and Ada district veterinary clinic and their link to rational drug use

Name of disease/cases diagnosis	Number of cases	Number of drugs prescribed			
		Antibiotics		Anthelmintics	
		Yes	No	Yes	No
Intestinal helminthosis	514	153^b^	361^a^	425^a^	89^b^
Ectoparasites^e^	204	109^b^	95^a^	93^ad^	111^c^
Respiratory diseases	117	87^a^	30^c^	54^ab^	63^ba^
Surgical cases	60	55^a^	5^c^	4^c^	56^a^
GIT disturbances^f^	22	11^c^	11^c^	6^c^	16^c^
Reproductive problems	29	15^c^	14^c^	4^b^	25^a^
Blackleg	86	73^a^	13^b^	22^c^	64^a^
Lumpy skin disease	51	49^a^	2^c^	4^c^	47^a^
Sheep pox disease	29	24^a^	5^c^	4^c^	25^a^
Anthrax	4	4^a^	0^a^	0^a^	4^a^
Others	63	0^a^	63^a^	20^a^	43^c^
Total cases tentatively diagnosed	1179	580		636	
Not adequately specified cases or diseases	782	636^c^	146^a^	233^c^	549^c^
Total	1961	1216		869	

*GIT* gastrointestinal tract

^a^Rational

^b^Irrational

^ab^Rational if the cause is due to lung worms

^ba^Rational if the cause is only due to bacterial diseases

^c^Difficult to categorize as rational/irrational as it depends on other co-infections

^d^The drug administered was ivermectin

^e^Mange mites, ticks and fleas

^f^Depends on the causative agents

Among all patients admitted to CVMA-VTH and Ada district veterinary clinic, 1757 (96.6 %) were treated empirically, without getting correct definitive (laboratory-supported) diagnosis. The remaining 62 (3.4 %) were diagnosed based on pathognomonic and specific clinical signs rather than confirmatory laboratory tests. The routes of drug administration were not written for 98.9 % (2210/2235) of the prescribed drugs.

The educational level and work experience of the prescribers were also assessed. One thousand two hundred eighty eight (70.8 %) and 531 (29.2 %) of the prescriptions were done by animal health assistants and veterinarians, respectively. Among all medicines prescribed, the majority (n = 1414, 77.7 %) was prescribed by professionals who have a work experience of >10 years, followed by less than 5 years (n = 312, 17.2 %) and between 5 and 10 years (n = 93, 5.1 %) of experiences.

**Fig. 1:**
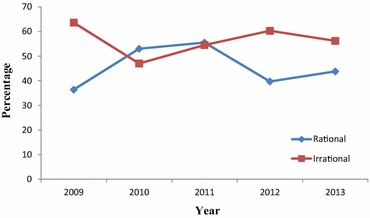
Trend of evaluation of rational drug prescription pattern by year at CVMA-VTH and Ada district veterinary clinic from 2009 to 2013

Chi Square test for trend analysis of rational use of veterinary drugs across the years showed a statistically significant association between irrational drug usage and year, with a peak irrational use in the year 2009 (p = 0.000). The percentage of irrational drug use across the years was 63.6 % (213/335) for 2009, 47.0 % (142/302) for 2010, 54.5 % (220/404) for 2011, 60.3 % (191/317) for 2012 and 56.2 % (259/461) for 2013 respectively (Fig. 1). The results showed insignificant reduction in trend of irrational drug use over the 5 years.

## Discussion

The average number of drugs per prescription at CVMA-VTH and Ada district veterinary clinic was 1.23, which indicates the absence of poly pharmacy. However, the WHO standard for humans is 1.6–1.8 [9, 18]. Although there is no study on veterinary drug prescription pattern, reports of studies performed on human subjects are available. For instances, the study done in southwest Ethiopia, Jimma Hospital, has shown that the average number of drugs per encounter was 1.59 [13]. Additionally, in other study conducted in three hospitals in north Ethiopia, the average number of drugs per patient was 0.98 at Gondar Hospital, 1.8 in Bahir Dar Hospital, and 2.2 in Debre Tabor Hospital [19]. A national baseline study done on drug use indicators of humans in Ethiopia in September 2002 showed the average number of drugs prescribed per encounter to be 1.9 [20]. The study of drug use patterns on human subject in 12 developing countries has also shown the average number of drugs per encounter was high in Nigeria (3.8), low in Sudan (1.4), and in Zimbabwe (1.3) [21]. A high average number of drugs on humans might be due to financial incentives to prescribers to prescribe more, lack of therapeutic training of prescribers, or shortage of therapeutically correct drugs. The low values might mean there is constraint in the availability of drugs, or prescribers have appropriate training in therapeutics [15]. However, the low value in our study indicates the absence of medicines in the clinic rather than belief of prescribers having appropriate training.

The percentage of drugs prescribed by generic name in the present study (90.1 %) is less than the standard derived to serve as ideal (100 %) [18]. A national baseline study on drug use indicators in Ethiopia in September 2002 also showed the percentage of drugs prescribed by generic name for human subjects was 87 % [12], which is lower than our finding of 90.1 %. In the study conducted in 12 developing countries (human subject), the percentage of generic drugs prescribed was low in Nigeria (58 %) and Sudan (63 %) but encouraging in Tanzania (82 %) and Zimbabwe (94 %) [21].

The percentage of encounters in which antibiotics and anthelmintics were prescribed at CVMA-VTH and Ada district veterinary clinic were 54.4 and 38.9 %, respectively. The ideal standard percentage of encounters in which antibiotics are prescribed for humans is 20.0–26.8 % [9, 18]. This finding suggests that antimicrobials and anthelmintics are over prescribed and needs to be regulated. The high percentage of antibiotics prescribed in this study setting may be due to inadequate recognition of the disease, unavailability of diagnostic aids for confirmatory tests, absence of a right drug, prescribers’ belief of the therapeutic efficacy of the antibiotics is low and prescribers’ knowledge. A national baseline study on drug use indicators (human subjects) in Ethiopia in September 2002 also showed that the percentage of encounters in which an antibiotic was prescribed to be 58.1 % [12], which was nearly similar to our finding.

Though the primary purpose of veterinary drugs is to safeguard the health and welfare of animals [22], 656/1216 (53.9 %) antimicrobials and 233/869 (26.8 %) anthelmintics were prescribed irrationally for 782 (43.0 %) cases not adequately diagnosed. Additionally, 27.5 % antimicrobials were also prescribed irrationally to treat diseases that were tentatively diagnosed as parasitic cases and to prevent secondary bacterial complications from viral diseases. Almost all the cases (96.6 %) that were encountered in CVMA-VTH and Ada district veterinary clinic received drug therapy after they had been tentatively diagnosed without getting correct laboratory-supported diagnosis. However, the routes of drug administration were not indicated for 98.9 % of the prescribed drugs, which reveals the presence of irrational drug use. The four main reasons of irrational antibiotic prescribing are inadequate recognition of infections that lead to prescription of unnecessary drugs, inappropriate choice of route, dose and duration of antibiotics [23].

A slowly declining trend of irrational drug prescription pattern was observed between 2009 (63.6 %) and 2013 (56.2 %) in this study (Fig. 1). This shows a promising result which may be attributed to the ongoing comprehensive efforts to strengthen the rational drug use policy of the country. This may also signify further intervention is required to create awareness among professionals to use and prescribe veterinary drugs rationally.

Drugs are the most frequently detected chemical residues in foods of animal origin, overwhelmingly majority of which are antimicrobials [24] commonly used in veterinary practice in this study. Drug residues in animal-derived food products are an important consideration for consumers. Residues of drugs used in the food-animal industry threaten human health by being acutely or cumulatively allergenic, toxic, mutagenic, teratogenic, or carcinogenic and may favour the emergence of resistant microbial strains within a host [25]. Antibiotic not recommended for use in food animals, for instances, chloramphenicol [26], one of antibacterial agents listed in essential medicines in both veterinary [16] and human [27] medicines for Ethiopia was also prescribed in this study. This drug is still used as a first line to treat anaplasmosis and brucellosis, and as second line for campylobacteriosis and endometritis in animals [28]. Besides, it is also used as a first line drug to treat typhoid fever and pyogenic meningitis infections, and as a second line agent to treat gastroenteritis infections in humans [29]. Residue of this drug is believed to be carcinogenic in humans and responsible for the emergence of resistant microbial strains [26].

The major considerations for proper usage of antimicrobial agents, which are a main concern of modern medicine, are to select the optimal agent at the proper dosage and duration, to minimize the emergence of resistance and to provide health services at a reasonable cost [30]. There is great evidence that antimicrobial agents are often abused and used excessively and in this study, 54.4 % (1216) of the prescriptions were irrational. Although a review done by Alemu et al. indicates that many infectious agents have developed resistance against oxytetracycline [31], this drug is the most prescribed antibiotics observed in this study (45.5 %), which is similar to a study on antimicrobial drug use in Kenya by Mitema et al. [32]. Overuse of antibiotics is an indication of a problem because it could facilitate the emergence of resistance bacterial strains [33]. Moreover, the cost incurred is high due to extravagant prescription where drugs are indicated for a viral infection or for an infection in which symptomatic treatment is enough. Albendazole and ivermectin, which are used for the treatment of parasitic diseases, are also commonly available and utilized at veterinary clinics. As a result, over use of these drugs might favour development of anthelmintic resistance [7] in the study area.

Ideally, good antibiotic prescribing practice should reflect the use of the most effective, least toxic, and least costly antibiotic for the precise duration of time needed to cure the infection [34, 35]. Unfortunately, according to the study conducted by Gyssens up to 40–60 % of the antibiotics were prescribed inappropriately [34], which is consistent with our findings (54.4 %). Subject to many underlying factors, the widely spread irrational use of veterinary drugs needs to be tackled through various interventions, including the introduction of guidance on the use of drugs. Though formulary for veterinary drugs is believed to be one of the key guiding instruments for the facilitation of the promotion of rational use [16], it was not used in both CVMA-VTH and Ada district veterinary clinic.

### Study limitation

Although the absences of previous studies on rational use of veterinary drugs and standards has created difficulties to make comparison, the study is the pioneer in attempting to indicate the presence of irrational veterinary drug usage in central Ethiopia. Routes of drug administration and duration of treatment were not fully specified for most cases. Some cases were not specified properly (e.g., respiratory diseases, GIT disturbances and reproductive problems) and mainly based on tentative diagnosis. Confirmatory laboratory tests were also not done for the majority of cases. As a result, evaluation of rational use of veterinary drugs, specially antimicrobials and anthelmintics, prescribed for various clinical cases or diseases were made mainly based on clinical signs and symptoms.

## Conclusions

The findings of the prescribing practices for veterinary drugs have showed that there were problems of correct diagnosis and the availability of both standard veterinary treatment guideline and drug formulary in the study area, which could lead to irrational drug use. On the other hand, polypharmacy and prescribing from EVDL were not found to be a problem. Researchers and policymakers to improve prescribing practice and rational drug use in veterinary medicine can use the baseline data gathered in the present study. The government, private animal health care institutions, individual animal health care providers and animal owners all have a responsibility to promote rational use of medicines. Hence, integrated national databases to support a rational, visible, science-driven decision-making process and policy development for regulatory approval and use of antimicrobials in food animals, which would ensure the effectiveness of these drugs and the safety of foods of animal origin, should be established.

## Abbreviations

CVMA-VTH: College of Veterinary medicine and agriculture veterinary teaching hospital
EVDL: Veterinary drug list of Ethiopia
GIT: gastrointestinal
WHO: World health organization
